# Multimodal Treatment With Nivolumab Contributes to Long-Term Survival in a Case of Unresectable Esophagogastric Junction Neuroendocrine Carcinoma

**DOI:** 10.7759/cureus.65981

**Published:** 2024-08-01

**Authors:** Shunya Hanzawa, Shinya Asami, Takashi Kanazawa, Satoshi Oono, Norihisa Takakura

**Affiliations:** 1 Department of Surgery, Fukuyama City Hospital, Fukuyama, JPN; 2 Department of Gastrointestinal Surgery, Okayama University Hospital, Okayama, JPN

**Keywords:** immune checkpoint inhibitor, conversion surgery, multidisciplinary therapy, nivolumab, esophageal neuroendocrine carcinoma

## Abstract

Advanced neuroendocrine carcinoma (NEC) has an extremely poor prognosis, partly explained by the rarity and diagnostic difficulty, for which the most appropriate treatment strategy has not been established. In this report, we discuss a case of unresectable advanced esophagogastric junction NEC, which was difficult to diagnose, that has achieved relatively long-term survival with multidisciplinary treatment centered on nivolumab. A man in his 60s was initially diagnosed with an advanced esophagogastric junction squamous cell carcinoma (SCC). The lymph node metastasis was detected in the regional lymph nodes and para-aortic region. We diagnosed the patient with T3, N3, M1 (Lym), stage IVB, and administered systemic chemotherapy. Due to the failure of first-line, fluorouracil, and cisplatin therapy, we administered nivolumab as the second-line therapy. This therapy demonstrated partial response, so we performed conversion surgery, however the postoperative diagnosis was NEC. Three years after treatment initiation, a single lymph node metastasis has recurred, which is under control with nivolumab and radiation therapy. However, 4.5 years after the start of treatment, with the advent of immune-related adverse events (irAE), nivolumab was discontinued and the patient was placed on surveillance. Six months after that, metastasis to the hilar lymph node and adrenal gland was observed. Both times that recurrence/metastasis appeared, they occurred while nivolumab was being discontinued, suggesting its significant systemic anti-cancer effect. Therefore, nivolumab in particular may be an effective treatment for advanced esophageal NEC, and this case suggests that it may contribute to prolonged progression-free survival.

## Introduction

Esophageal and gastric neuroendocrine carcinoma (NEC) are rare tumors with an incidence of 0.4%-2% and 0.1%-0.6%, respectively, and have an extremely poor prognosis [1,2]. It is highly aggressive and frequently associated with metastasis to the lymph nodes and distant organs at an early stage, with a reported five-year survival rate of 9% and median survival of 4.2-18.5 months [3]. For unresectable gastrointestinal NEC, first-line treatment with a combination of cisplatin and etoposide or irinotecan is recommended. However, there is currently no established treatment for subsequent lines of therapy [4]. This poor outcome could be partly explained by the rarity and diagnostic difficulty of esophageal-gastric NEC, and the fact that the most appropriate treatment strategy has not been established.

On the other hand, based on the results of the ATTRACTION-3 trial [5], nivolumab is recommended as the second-line therapy for unresectable advanced or recurrent esophageal cancer in Japan. In this regard, nivolumab demonstrated superior overall survival compared to the conventional standard treatment, such as paclitaxel or docetaxel monotherapy. Furthermore, as first-line treatment for unresectable advanced or recurrent esophageal cancer, immune checkpoint inhibitors (ICIs) such as pembrolizumab and ipilimumab are increasingly used due to the accumulated findings from the KEYNOTE-590 and Checkmate-648 trial [6,7], in actuality, the former is recommended in the Japanese treatment guideline 2022 [8].

In this case, the initial diagnosis was unresectable esophageal-gastric junction squamous cell carcinoma (SCC), and fluorouracil and cisplatin therapy were introduced as the first-line treatment, but disease progression was observed. Therefore, nivolumab as a second-line treatment was introduced, and it proved effective, allowing for conversion surgery. However, a pathological diagnosis after surgery revealed NEC, uncovering an initial diagnostic error. Nevertheless, the patient achieved relatively long-term survival through multidisciplinary treatment.

Herein, we discuss the diagnostic challenges due to the rarity of unresectable advanced esophageal-gastric junction NEC, and the potential utility of multidisciplinary treatment, including drug therapy, surgery, radiation therapy, and particularly nivolumab.

## Case presentation

**Figure 1:**
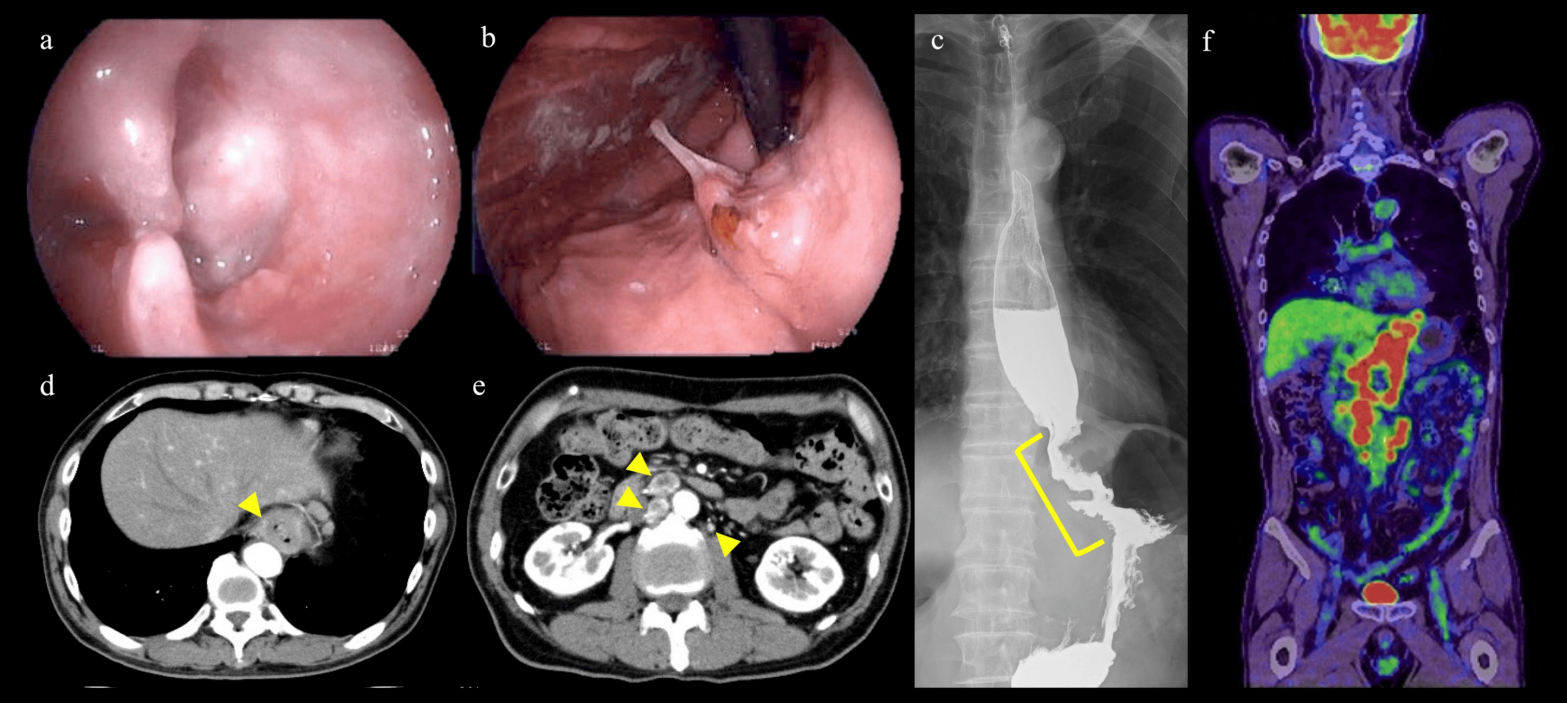
Preoperative images. (a, b) Upper gastrointestinal endoscopy showing an ulcerative and infiltrative advanced esophageal tumor with circumferential stenosis extending from the abdominal esophagus to the cardia. (c) Upper gastrointestinal tract radiography showing an irregular mucosa with stenosis of 5 cm in length from the abdominal esophagus to the cardia. (d, e) CT showing esophageal wall thickening extending from the abdominal esophagus to the hilum, along with enlarged lymph nodes in the lower para-esophagus, cardiac region, lesser curvature of the stomach, and para-aortic region (yellow arrowhead). (f) PET-CT showing abnormal fluorodeoxyglucose (FDG) accumulation in the primary lesion, and lymph nodes in the lower para-esophagus, cardiac region, lesser curvature of the stomach, and para-aortic region. No obvious distant organ metastasis was detected.

A 60-year-old man was referred to our hospital after an upper gastrointestinal endoscopy revealed an ulcerative and infiltrative type tumor at the esophagogastric junction (Figure 1a, 1b). The results of the tissue biopsy at his previous physician showed a large infiltration of cells with a high n/c ratio and a lack of keratinizing tendency, although a poorly differentiated SCC was suspected, taking into account the frequency of the disease. The upper gastrointestinal series showed an irregular mucosa with stenosis extending from the abdominal esophagus to the cardia for approximately 5 cm (Figure 1c). Thoracic and abdominal contrast-enhanced computed tomography (CT) showed no unresectable factors associated with local progression; however, there were some swollen lymph nodes in the lower para-esophagus, cardiac region, lesser curvature of the stomach and para-aortic lymph nodes in the extra-regional lymph nodes (Figure 1d, 1e). In addition, positron emission tomography (PET)-CT showed abnormal fluorodeoxyglucose (FDG) accumulation (SUV-Max 14.28) in the primary lesion, the regional lymph nodes mentioned above, and the para-aortic lymph nodes (Figure 1f). There was no other distant organ metastasis. The serum SCC antigen and carcinoembryonic antigen (CEA) levels were within the normal range at 0.9 ng/mL and 4.2 ng/mL, respectively. According to these results, the preoperative diagnosis was esophagogastric junction cancer, Siewert type Ⅰ, T3, N3, M1 (Lym), stage IVB (the Union for International Cancer Control 8th Edition [9]). At this stage of progression, surgery alone would not be curative; therefore, we decided to administer systemic chemotherapy.

Chemotherapy

**Figure 2:**
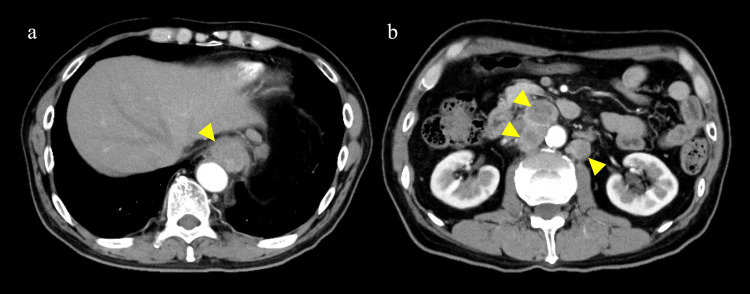
Post-treatment images (after two courses of fluorouracil and cisplatin therapy). (a, b) CT showing that both the primary lesion and metastatic lymph nodes tended to increase in size (yellow arrowhead).

Fluorouracil (800 mg/m^2^) + cisplatin (80 mg/m^2^) was administered as the first-line chemotherapy. Post-treatment CT showed that both the primary tumor and the metastatic lymph nodes tended to increase in size (Figure 2a, 2b). Additionally, the serum CEA was elevated to 10.6 ng/mL.

**Figure 3:**
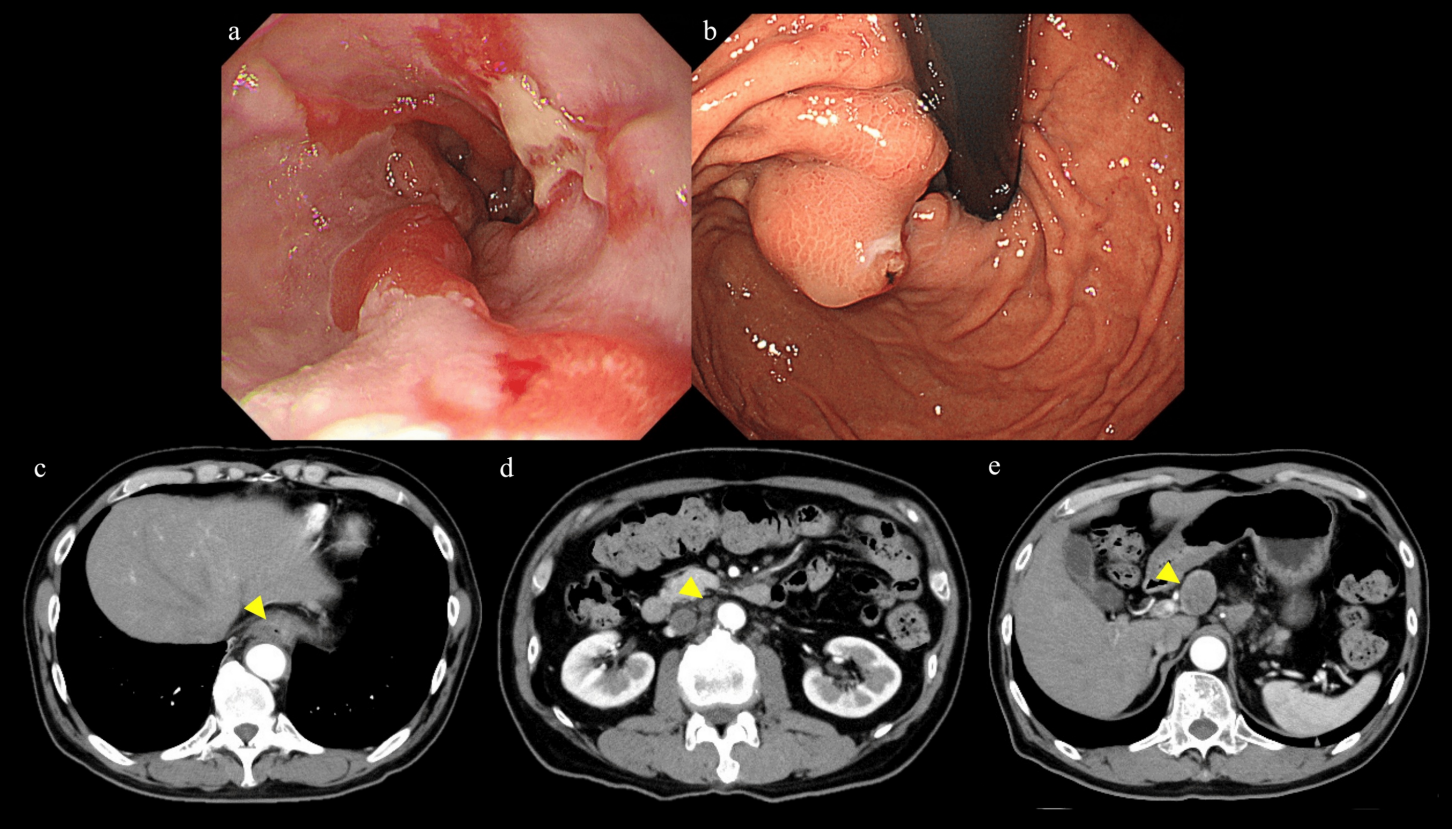
Post-treatment images (after eight courses of nivolumab therapy). (a, b) Upper gastrointestinal endoscopy showing mild shrinkage of the tumor. (c, d) CT showing that both the primary lesion and metastatic lymph nodes tended to shrink in size (yellow arrowhead). (e) Some lymph nodes (in the region of the anterior superior portion of the common hepatic artery) showed a tendency to increase in size (yellow arrowhead).

Due to disease progression, we selected nivolumab (240 mg/body) as the second-line therapy. After eight courses of nivolumab, the primary tumor and metastatic lymph nodes showed a partial response, and his serum CEA level also decreased to the normal range of 3.5 ng/mL. Several lymph nodes (especially in the anterior superior portion of the common hepatic artery) showed mild enlargement (Figure 3a-3e); however, unresectable factors disappeared and downstaging (yT2, yN2, yM0) was achieved, so conversion surgery was performed.

Conversion surgery

**Figure 4:**
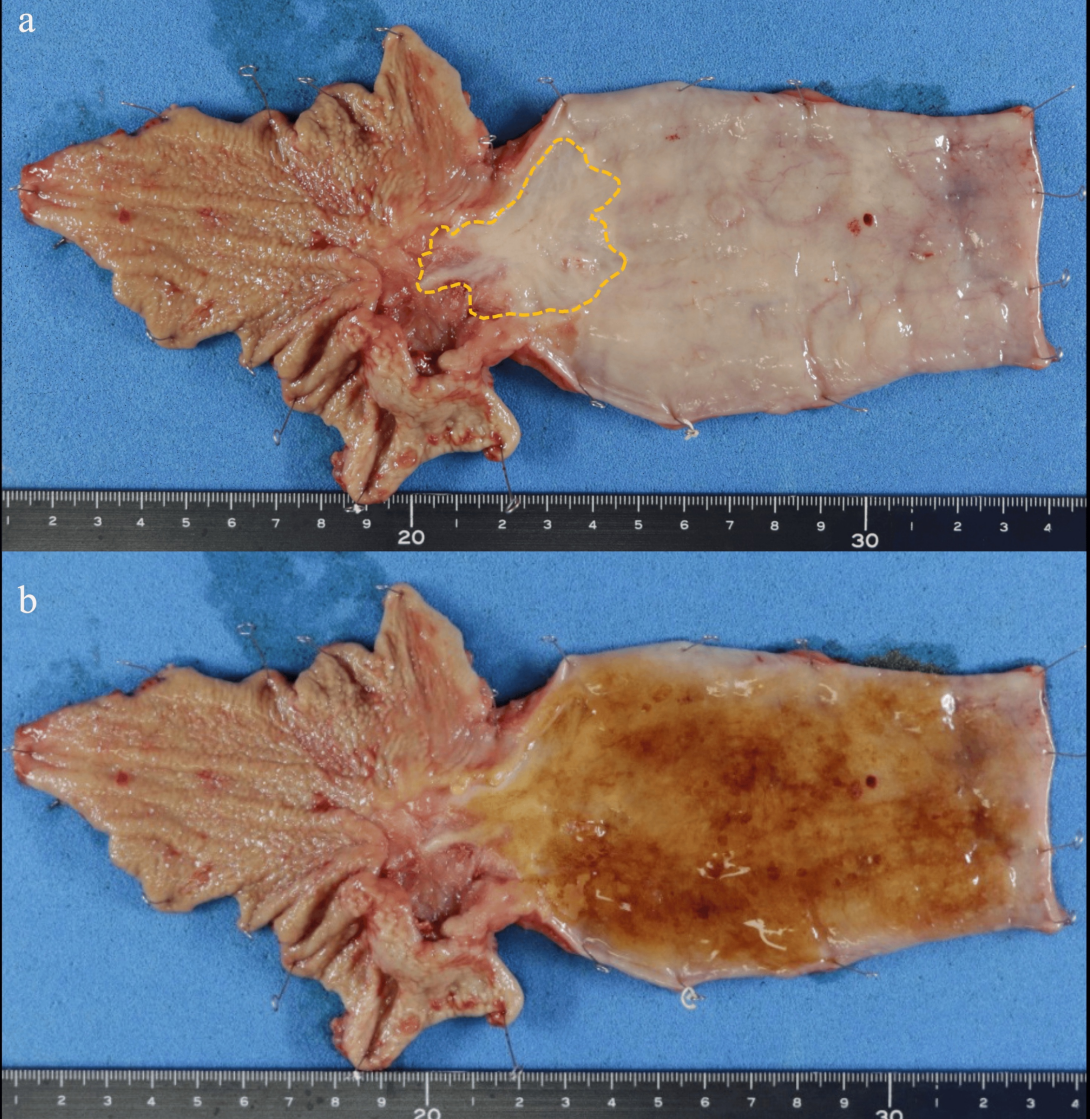
Macroscopic findings of the resected specimen. (a) Ulcerative lesion at the esophagogastric junction, suspected tumor remnant (yellow dotted line). (b) The tumor was not noticeably unstained with Lugol staining.

We performed subtotal esophagectomy, along with lymph node dissection of two lesions, para-aortic lymph node dissection, and gastric conduit reconstruction using the posterior mediastinal route. The excised specimen is shown in Figure 4. The patient was discharged from the hospital on postoperative day 20 without any complications.

Histopathological diagnosis

**Figure 5:**
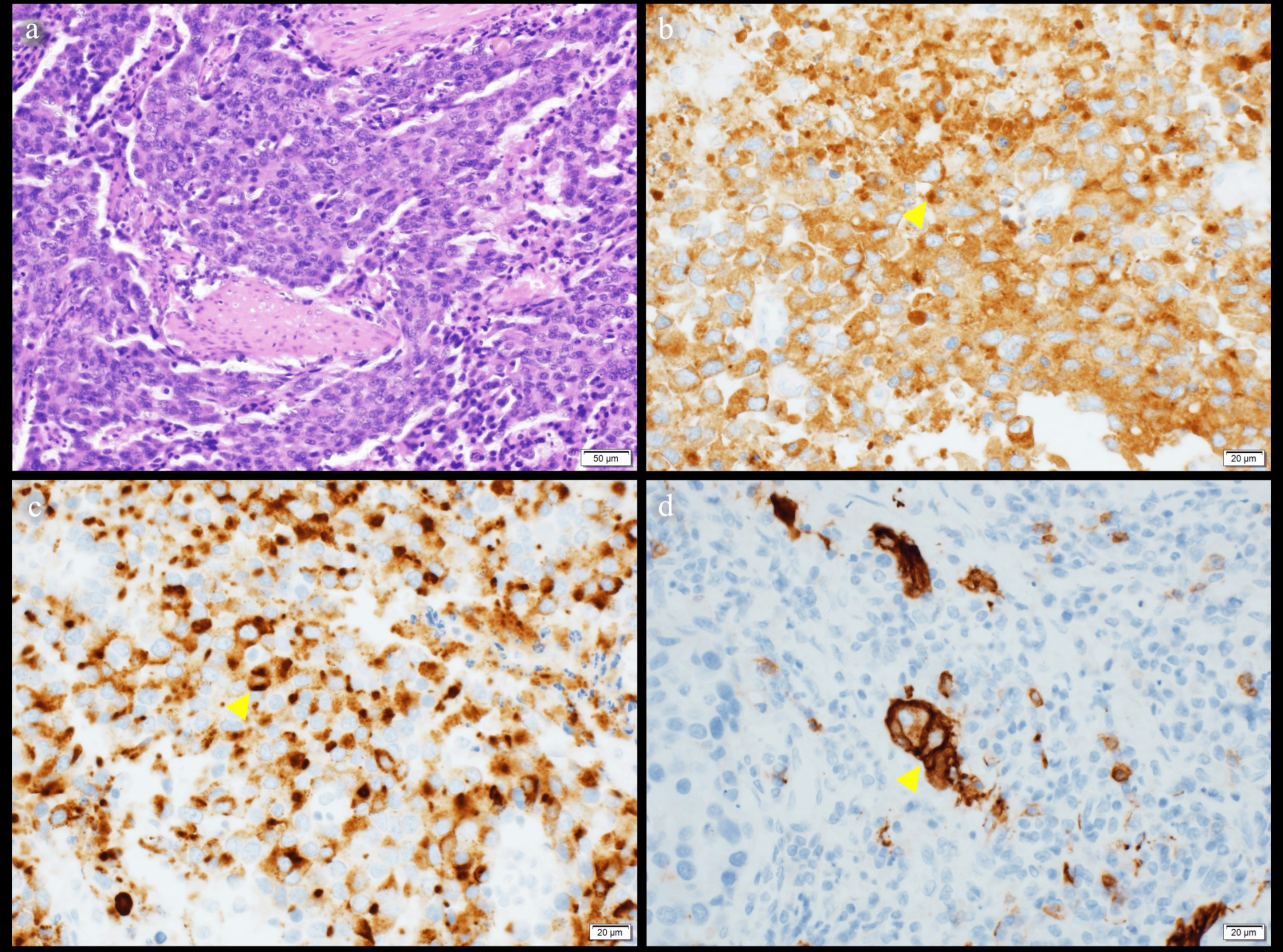
Microscopic findings of the resected specimen. (a) Hematoxylin and eosin staining showing small, atypical cells with a high N/C ratio infiltrating and proliferating in the shape of a full nodule. (b) Immunostaining for synaptophysin was positive (yellow arrowhead). (c) Immunostaining for chromogranin A was positive (yellow arrowhead). (d) Immunostaining for CD56 was positive (yellow arrowhead).

The histopathological findings of the resected specimens are shown in Figure 5. Hematoxylin and eosin staining revealed that small, atypical cells with a high N/C ratio infiltrated and proliferated in the shape of a full nodule (Figure 5a). On the immunohistochemistry (IHC), the specimens demonstrated positive synaptophysin, chromogranin A, and CD56, with a Ki-67 labeling index of 79% (Figure 5b-5d). We diagnosed NEC. Histological evaluation of the response to treatment showed that most of the dissected lymph nodes were fibrotic scar tissue. However, there were viable NEC cell remnants in the primary tumor and lymph nodes of the lesser curvature of the stomach, the left gastric artery trunk, and the anterior superior portion of the common hepatic artery.

Radiation therapy and clinical course

**Figure 6:**
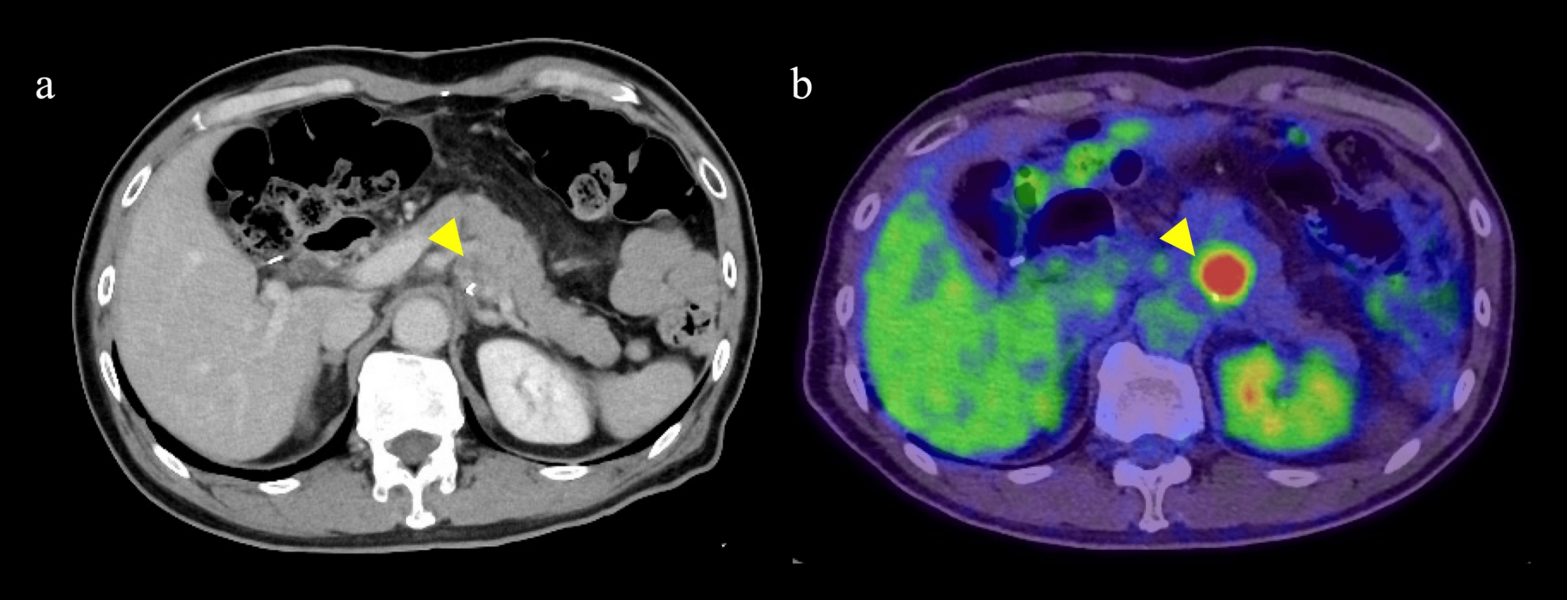
Images of recurrent lymph nodes. (a) Enlarged lymph nodes on the ventral side of the superior mesenteric artery (yellow arrowhead). (b) PET-CT shows abnormal fluorodeoxyglucose (FDG) accumulation in the same region (yellow arrowhead).

No adjuvant chemotherapy was administered, and during follow-up (at 10 months after surgery), a single lymph node recurrence along the superior mesenteric vein was observed (Figure 6a, 6b). Therefore, nivolumab and radiation therapy were simultaneously introduced (55 Gy delivered in 22 fractions to the local).

**Figure 7:**
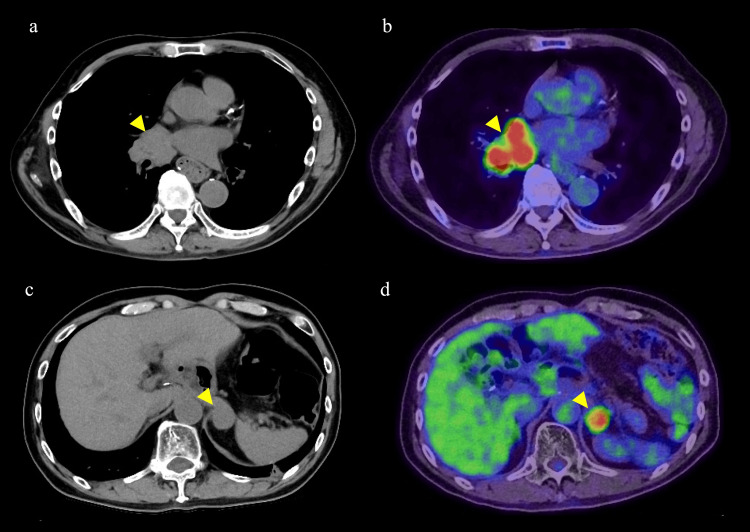
Images of distant organ recurrence. (a) CT shows swelling of the right hilar lymph nodes (yellow arrowhead). (b) PET-CT shows abnormal fluorodeoxyglucose (FDG) accumulation in the same region (yellow arrowhead). (c) CT shows a mass in the left adrenal gland (yellow arrowhead). (d) PET-CT shows abnormal FDG accumulation consistent with the mass (yellow arrowhead).

After completion of radiation therapy, nivolumab was continued alone, and this lesion has remained shrunken and is under surveillance with no additional recurrent lesions. However, at the time of 35 courses, bullous pemphigoid appeared, so it was judged as immune-related adverse events (irAE), and nivolumab was discontinued and the patient was followed up. Six months after withdrawal of nivolumab (five years after the start of treatment), metastasis to the right hilar lymph node and left adrenal gland was observed (Figure 7a-7d), and radiation therapy (50 Gy delivered in 20 fractions to the local) was performed, the patient is still alive.

## Discussion

We experienced a case of esophagogastric junction NEC that achieved long-term survival with multimodal treatment including nivolumab. As mentioned above, NEC is challenged by the rarity of the disease itself, the difficulty of its diagnosis, and the lack of established treatment strategies [1-3]. 

PubMed was searched for articles published in English using the terms "neuroendocrine carcinoma" and "nivolumab"; 41 articles were retrieved, but only a few included esophageal and gastric NEC. The median overall survival of patients with the primary site in the esophagus and stomach was 13.4 and 13.3 months, respectively, suggesting a severely poor prognosis [10]. Some report that multidisciplinary treatment, including surgical resection, improves prognosis even in advanced cancer [11-12]. The 2016 European Neuroendocrine Tumor Society consensus guidelines recommend platinum-based regimens such as cisplatin plus irinotecan or etoposide as primary treatment for gastrointestinal NEC, however, response rates are not sufficient and there are currently no recommended agents for secondary treatment [4]. Unfortunately, there is a lack of evidence supporting the efficacy of ICIs for NEC. 

In this case, we would like to discuss two points. The first concerns the error in the initial diagnosis and the failure of the first-line treatment. The initial diagnosis was SCC, and NEC could not be diagnosed on the preoperative biopsy specimen and was only confirmed on examination of the postoperative resection specimen. Additional IHC was performed on the lymph nodes to evaluate pathological tumor remnants, and the residual tumor component was also confirmed to be NEC. However, in the field of esophageal and gastric cancer, there is intra-tumor heterogeneity and the proportion and location of NEC components in the primary tumor differ between tumors [13]. Similar to the present case, there are some reports of NEC components being detected in postoperative resection specimens and lymph nodes, suggesting the difficulty of preoperative diagnosis [13,14]. Preoperative diagnosis is made in less than 20% of cases, and Imamura et al. report that the complex type is first identified in the resection specimen [15]. While we considered performing this analysis postoperatively, this was not possible because the specimen block did not remain with the referred clinic. This suggests the importance of performing IHC on the initial biopsy specimens, and we cannot rule out that errors in initial diagnosis may have influenced the choice of first-line treatment and disease progression.

The second is that nivolumab was the key drug in this case. First, from a histological aspect, we added a search for microsatellite instability (MSI) status and PD-L1 expression, which could be examined at our hospital postoperatively. MSI status was microsatellite stable and PD-L1 has a combined positive score (CPS) ≥10. Since PD-L1 status of CPS ≥10 is an indication for ICIs (i.e., pembrolizumab) in esophageal SCC or adenocarcinoma, this factor may have contributed to the success of this NEC patient. Next, from a clinical perspective, it played a very important role in the response to second-line therapy and in disease control for recurrent lesions. Specifically, it shrank the tumor and metastatic lymph nodes enough to allow conversion surgery and contributed to disease control of extremely aggressive NEC for approximately three years. Both times that recurrence/metastasis appeared, they occurred while nivolumab was being discontinued, suggesting its significant systemic anti-cancer effect.

The combination of radiotherapy and nivolumab for recurrent disease has been reported by Voronova et al. and Takagi et al. and was followed in this case [16,17]. Although no definite evidence exists and the tumor microenvironment, such as infiltration of CD8-positive T cells in the specimen, has not been evaluated, it is possible that activation of the immune profile of the tumor microenvironment with induction of immunogenic cell death may be involved. These findings suggest that the high expression of PD-L1 in tumors for preoperative treatment and the increased immunogenicity of tumors by radiation therapy for the treatment of recurrent lesions may have contributed to the efficacy of ICI, respectively.

In recent years, with the increasing use of ICIs in the field of esophageal and gastric cancer, the likelihood of encountering an incidental NEC case like this one may increase. Clinical trials of ICIs for neuroendocrine neoplasms (NEN) are also underway. In this regard, the phase II basket trial of dual anti-CTLA-4 and anti-PD-1 blockade in rare tumors (DART SWOG 1609) reported that the combination of ipilimumab and nivolumab was well tolerated in patients with high-grade, regardless of the primary site, with an overall response rate (ORR) of 42% [18]. According to the exploratory analysis of CheckMate-026 [19], the ORR and progression-free survival of the nivolumab group was better in patients with high tumor mutational burden (TMB), suggesting that ICIs might be effective in the high TMB group. Furthermore, Capdevila et al. reported the safety and modest survival benefit of a dual checkpoint blockade of durvalumab plus tremelimumab as second-line therapy for advanced NEN [20]. As mentioned above, cross-organ research is underway in this field, and we are waiting for further accumulation of cases and findings. 

## Conclusions

We have experienced a case in which multimodal treatment with nivolumab did not achieve a complete response but resulted in relatively long-term survival. This case is suggestive in two ways: firstly, it underscores the importance of initial diagnosis given the rarity of the disease; and secondly, although the findings were incidental, nivolumab could be a potential treatment option for esophagogastric neuroendocrine carcinoma (NEC). Additionally, multimodal therapy including immune checkpoint inhibitors may be an effective treatment option for patients with advanced esophagogastric NEC.
